# Identification and Validation of an Annexin-Related Prognostic Signature and Therapeutic Targets for Bladder Cancer: Integrative Analysis

**DOI:** 10.3390/biology11020259

**Published:** 2022-02-07

**Authors:** Xitong Yao, Xinlei Qi, Yao Wang, Baokun Zhang, Tianshuai He, Taoning Yan, Lu Zhang, Yange Wang, Hong Zheng, Guosen Zhang, Xiangqian Guo

**Affiliations:** 1 Cell Signal Transduction Laboratory, Department of Predictive Medicine, Institute of Biomedical Informatics, Bioinformatics Center, Henan Provincial Engineering Center for Tumor Molecular Medicine, School of Basic Medical Sciences, Academy for Advanced Interdisciplinary Studies, Henan University, Kaifeng 475004, China; 104753190854@henu.edu.cn (X.Y.); 104753190845@henu.edu.cn (X.Q.); 104753201156@henu.edu.cn (Y.W.); hts@henu.edu.cn (T.H.); 104753211246@henu.edu.cn (T.Y.); 10190146@vip.henu.edu.cn (L.Z.); wangyange3@henu.edu.cn (Y.W.); zhhong@henu.edu.cn (H.Z.); 2 Beijing Key Laboratory of New Molecular Diagnosis Technologies for Infectious Diseases, Department of Biotechnology, Beijing Institute of Radiation Medicine, Beijing 100850, China; kunbzh0201@163.com

**Keywords:** bladder cancer, Annexin family, survival analysis, prognostic signature, therapeutic target

## Abstract

**Simple Summary:**

Identification of new prognostic biomarkers and therapeutic targets could be essential ways to improve the outcome of bladder cancer (BC) patients. In this study, we comprehensively analyzed the mRNA expression and prognosis of Annexin family members (ANXA1-11, 13) in BC through public analysis tools, including Oncomine, GEPIA2 and our in-house OSblca web server, and found that several Annexins were aberrantly expressed and associated with prognosis in BC. Then, we constructed and validated an Annexin-related prognostic signature (ARPS) in four individual BC cohorts through LASSO and COX regression, indicating that ARPS was an independent prognostic factor for BC. Briefly, our study was to determine the clinical significance of Annexins and provided a potential prognostic model and potential therapeutic targets for BC.

**Abstract:**

Abnormal expression and dysfunction of Annexins (ANXA1-11, 13) have been widely found in several types of cancer. However, the expression pattern and prognostic value of Annexins in bladder cancer (BC) are currently still unknown. In this study, survival analysis by our in-house OSblca web server revealed that high *ANXA1/2/3/5/6* expression was significantly associated with poor overall survival (OS) in BC patients, while higher *ANXA11* was associated with increased OS. Through Oncomine and GEPIA2 database analysis, we found that *ANXA2/3/4/13* were up-regulated, whereas *ANXA1/5/6* were down-regulated in BC compared with normal bladder tissues. Further LASSO analysis built an Annexin-Related Prognostic Signature (ARPS, including four members *ANXA1/5/6/10*) in the TCGA BC cohort and validated it in three independent GEO BC cohorts (GSE31684, GSE32548, GSE48075). Multivariate COX analysis demonstrated that ARPS is an independent prognostic signature for BC. Moreover, GSEA results showed that immune-related pathways, such as epithelial–mesenchymal transition and IL6/JAK/STAT3 signaling were enriched in the high ARPS risk groups, while the low ARPS risk group mainly regulated metabolism-related processes, such as adipogenesis and bile acid metabolism. In conclusion, our study comprehensively analyzed the mRNA expression and prognosis of Annexin family members in BC, constructed an Annexin-related prognostic signature using LASSO and COX regression, and validated it in four independent BC cohorts, which might help to improve clinical outcomes of BC patients, offer insights into the underlying molecular mechanisms of BC development and suggest potential therapeutic targets for BC.

## 1. Introduction

Bladder cancer (BC) is one of the most common malignancies with high risk of tumor recurrence and fatality in the urinary system. According to Global Cancer Statistics 2020, there were about 573,000 new cases and 212,000 deaths of BC around the world [1]. Although the significant advances in understanding of the underlying biology of BC have improved the accuracy and effect of diagnosing and treating this disease in recent years, BC still represents a spectra of diseases from recurrent noninvasive tumors to aggressive or advanced-stage disease that requires multimodal and invasive treatment [2,3]. Frequent postoperative recurrence and distant metastasis lead to the poor prognosis in BC patients [4,5]. Identification of efficient therapeutic targets, as well as new prognostic biomarkers are needed to improve the outcomes of BC patients.

Annexins belongs to a superfamily of calcium-dependent phospholipid-binding proteins and contains 12 members (ANXA1-11, 13) [6]. In eukaryotic cells, Annexins are involved in membrane trafficking and organization, such as vesicle transport, signal transduction, cell proliferation, cell differentiation and apoptosis [7,8]. Recent studies found that abnormal expression and dysfunction of Annexin proteins commonly occurred in tumor tissue and indicated that the disordered Annexin proteins may play important roles in tumorigenesis and progression, as well as chemoresistance in several types of cancer [9,10]. However, few studies reported the roles of Annexins in the carcinogenesis and prognosis in BC. Yu et al. found that the expression of ANXA1 was related to disease-free survival in BC patients and can be used as a recurrence biomarker for BC [11]. In addition, ANXA2 has also been found to play a key role in the formation, progression and recurrence of BC [12], and high expression of ANXA10 is significantly correlated with poor progression-free survival in BC patients [13]. However, the roles and mechanisms of most Annexins in BC remain unclear. 

In this study, we comprehensively analyzed the mRNA expression and prognosis of Annexin family members in BC through public analysis tools, including Oncomine, GEPIA2 and our in-house OSblca web server, and found that several Annexins were aberrantly expressed and associated with prognosis in BC. Then, we collected four BC datasets, including 703 BC samples with survival information from The Cancer Genome Atlas (TCGA) and Gene Expression Omnibus (GEO), and constructed and validated an Annexin-related prognostic signature (ARPS) in four individual BC cohorts through LASSO and COX regression, indicating that ARPS was an independent prognostic factor for BC. In addition, we further explored the biological functions and relevant pathways of ARPS through gene set enrichment analysis (GSEA), and analyzed the correlation between ARPS and the infiltrating immune cells using ssGSEA. Briefly, our study was to determine the clinical significance of Annexins and provided a potential prognostic model and potential therapeutic targets for BC. 

## 2. Materials and Methods

### 2.1. Survival Analysis of Annexin Family Members in OSblca 

OSblca (http://bioinfo.henu.edu.cn/BLCA/BLCAList.jsp, accessed on 2 December 2020) [14] is our in-house online survival analysis tool providing 1075 BC gene expression profiles and accompanied patient clinical follow-up information from TCGA and GEO databases. In OSblca, four types of survival endpoints, including overall survival (OS), disease-specific survival (DSS), disease-free interval (DFI), and progression-free interval (PFI) were provided for prognosis analysis. Each member of the Annexin family was analyzed for the relationship between their mRNA expression and BC outcomes in OSblca prognostic values of these genes were evaluated in all cohorts and survival terms, and, all cutoff values in ‘splitting the patients’ were tested in each cohort to get the best cutoff value.

### 2.2. Differential Expression Analysis of Annexin Family Members between BC and Adjacent Tissue by Oncomine and GEPIA2

Oncomine (www.oncomine.org, accessed on 2 December 2020) [15] and GEPIA2 (http://gepia2.cancer-pku.cn/#index, accessed on 2 December 2020) [16] databases were used to analyze the differential expression of Annexins between cancer and adjacent normal tissues. Oncomine is an online database that provides differentially expressed gene analysis using public microarray datasets. In Oncomine, mRNA expression of Annexin members between cancer tissue and adjacent normal tissue were compared with the thresholds of *p*-value < 0.05, |log2 (fold-change)| > 1, and the gene rank percentage < 10%. GEPIA2 provided the gene expression analysis based on TCGA and GTEx data. In GEPIA2, the expression of Annexins were compared between 404 bladder cancer samples and 28 normal samples with the threshold for *p*-value < 0.05 and |log2(fold-change) | > 1. In addition, differential expression of Annexins members in distinct clinical stages was also analyzed in TCGA bladder cancer samples using TISIDB database (http://cis.hku.hk/TISIDB/, accessed on 3 December 2020) [17].

### 2.3. Construction and Validation of the Annexin-Related Prognostic Signature through LASSO

Four individual BC cohorts with both gene expression data and related clinical follow-up information were downloaded from TCGA and GEO databases, including one TCGA BC dataset [18] (Discovery cohort) and three GEO BC datasets (Validation cohorts, GSE31684 [19], GSE32548 [20], GSE48075 [21]). Detailed information of each BC cohort was summarized in Supplementary Table S1. The work-flow was illustrated in Supplementary Figure S1. The ARPS was constructed using least absolute shrinkage and selection operator (LASSO) Cox regression through R package “glmnet”. The optimal parameter was determined through 10-fold cross validation with “family = cox, alpha = 1”, and with all other parameters set to default. Ultimately, ARPS is developed according to the following risk score formula:

$$\mathrm{risk}\;\mathrm{score}=\sum_{i}^{n}\left(\mathrm{Coefi}*\mathrm{Expri}\right)$$

where Coef_i_ is the coefficient of gene i in LASSO and Expr_i_ is the FPKM value of the included gene i.

Best cut-off risk score was calculated by using the “surv_cutpoint” function of R package “survminer” (https://CRAN.R-project.org/package=survminer, accessed on 24 December 2020). According to the best cut-off risk score, TCGA BC patients were divided into high- and low-risk groups and the prognosis between the two groups was evaluated through Kaplan-Meier survival analysis with the log-rank test. In addition, the expression heatmap of each Annexin member in ARPS and the risk score distribution and survival of patients were visualized through “pheatmap” package. Similar analyses were performed in three individual BC cohorts (GSE31684, GSE32548, GSE48075) to validate the prognosis performance of ARPS in BC.

### 2.4. Independent Prognostic Performance Analysis of ARPS in BC Cohorts

In the TCGA BC cohort, univariate Cox regression models were used to identify the prognostic clinical characteristics related to prognosis, and subsequently these significantly prognostic factors were further tested their independent prognostic performance through multivariate Cox regression models. Similar analyses were performed in other three individual BC cohorts (GSE31684, GSE32548, GSE48075) to validate the independent prognostic performance of ARPS in BC. 

### 2.5. Association between ARPS and Clinicopathology

The chi-squared test was performed to determine the association of clinical features between and ARPS in BC patients, where a *p*-value less than 0.05 indicates statistical significance. In addition, to verify the predictive effectiveness of ARPS in different subgroups, Kaplan-Meier survival analysis was used to compare the prognostic capability between subgroups in certain clinical features including age, gender, grade, lymph invasion status, T status, M status, and N status, TNM stage, and race.

### 2.6. Gene Interaction and Biological Functions of ARPS 

GeneMANIA was used to analyze the protein interactions between Annexin members in ARPS. To evaluate the biological functions of ARPS, differentially expressed genes (DEGs) between the two ARPS risk groups were identified through limma R package, and then were analyzed in the DAVID database to predict the gene ontology (GO) function and KEGG pathway. Furthermore, GSEA was implemented to reveal the potential mechanism that ARPS was involved in. 

### 2.7. Correlation between ARPS Risk Score and Imune Cell Infiltration and Immune Checkpoint Genes

In order to characterize the immune cell infiltration in the tumor microenvironment of two ARPS risk groups, the immune cell abundance of the TCGA BC cohort was calculated by estimate, timer, MCPcounter and xCell algorithm, and visualized through the “pheatmap” package of R software. Correlations between ARPS risk score and different immune cell abundances and immune checkpoint genes were analyzed through Pearson coefficient analysis. Then, the levels of immune cell infiltration and immune checkpoint gene expression between high- and low-risk groups were compared using the ‘limma’ package, which revealed the effect of ARPS risk score on BC immune microenvironment.

### 2.8. Statistical Analysis

Statistical analysis was performed using SPSS 16.0 and GraphPad Prism 5.0 software. Differences were compared by the Student’s t test or one-way analysis of variance (ANOVA) where appropriate. Statistical significance was determined by *p*-value less than 0.05.

## 3. Results

### 3.1. Survival and Differential Analysis of Annexins in Bladder Cancer 

**Figure 1:**
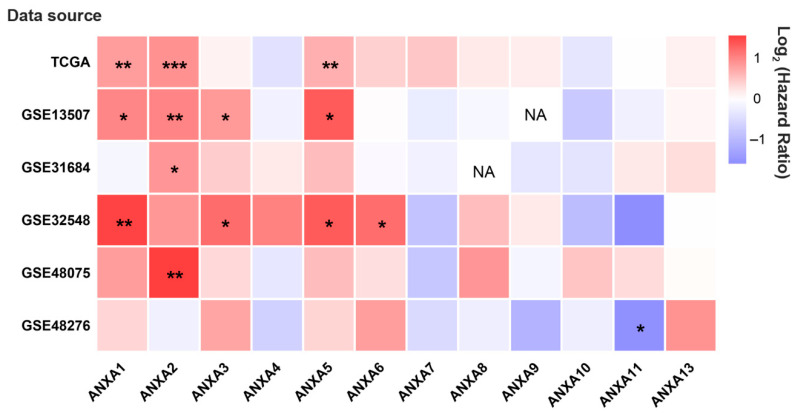
The hot map of prognostic value of Annexin family members regarding overall survival (OS) in BC patients by OSblca web server. Where * *p* < 0.05, ** *p* < 0.01 and *** *p* < 0.001, NA means the queried gene was non-existent in the dataset.

**Figure 2:**
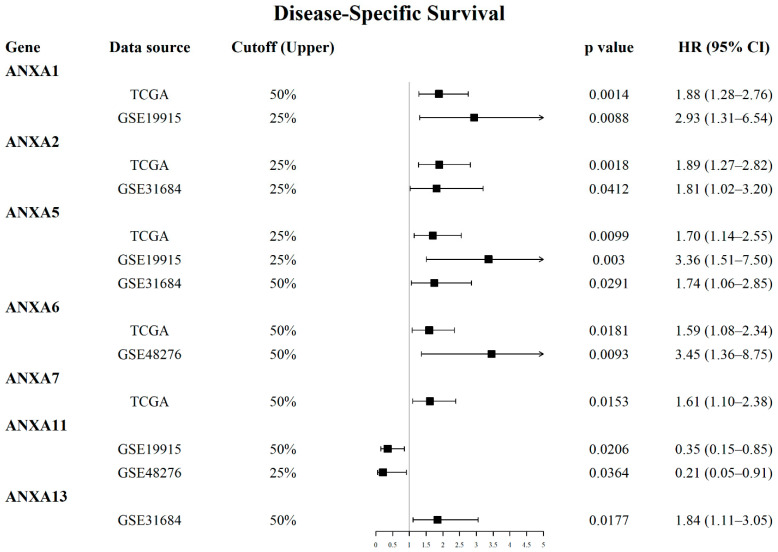
Forest plots displayed prognostic value of Annexin family members regarding disease-specific survival (DSS) in BC patients using OSblca web server.

**Figure 3:**
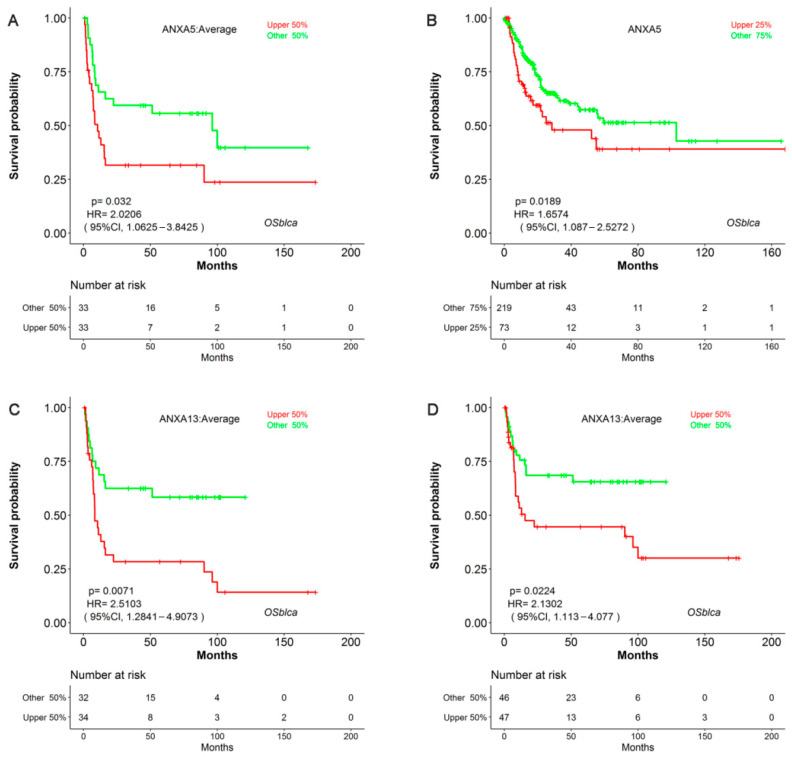
Survival analysis of *ANXA5* and *ANXA13* in disease-free interval (DFI) and progression-free interval (PFI) in BC patients using OSblca web server. Kaplan-Meier plotter of *ANXA5* with DFI (**A**) and PFI (**B**); Kaplan-Meier plotter of *ANXA13* with DFI (**C**) and PFI (**D**) in OSblca.

Survival analysis results revealed that the mRNA expression of more than half of Annexin members were related to the prognosis of BC patients. As shown in Figure 1, BC patients with high expression of *ANXA1/2/3/5/6* had a shorter OS time in comparison to those with low expression of *ANXA1/2/3/5/6*, while BC patients with high expressed *ANXA11* had a longer OS time. In particular, upregulated *ANXA1/2/5* were significantly associated with poor prognosis of BC in three or more datasets. In addition, the mRNA expressions of Annexins were also related to DSS in BC patients (Figure 2). The results indicated that BC patients with high expression of *ANXA1/2/5/6/7/13* showed a shorter DSS time than those with low expression, as opposed to the patients with high expression of *ANXA11*. Moreover, high expression of *ANXA5* and *ANXA13* were found to be associated with poor DFI and PFI in the BC cohort (Figure 3), indicating that these two genes might be involved in recurrence and progression of BC. 

Using the Oncomine database (Table 1), we found that most Annexins were significantly differentially expressed between BC and adjacent normal tissues. Markedly lower expressions of *ANXA1/5/6* were found in BC tissues consistently, while the expression levels of *ANXA2/3/4/13* were significantly increased in multiple BC cohorts. In the GEPIA2 database, *ANXA6* was significantly downregulated in TCGA BC samples compared to normal samples (*p*-value < 0.05), while *ANXA8* was significantly upregulated in TCGA BC samples, and no significant differences were found for other Annexins in BC (Supplementary Figure S1). 

**Figure 4:**
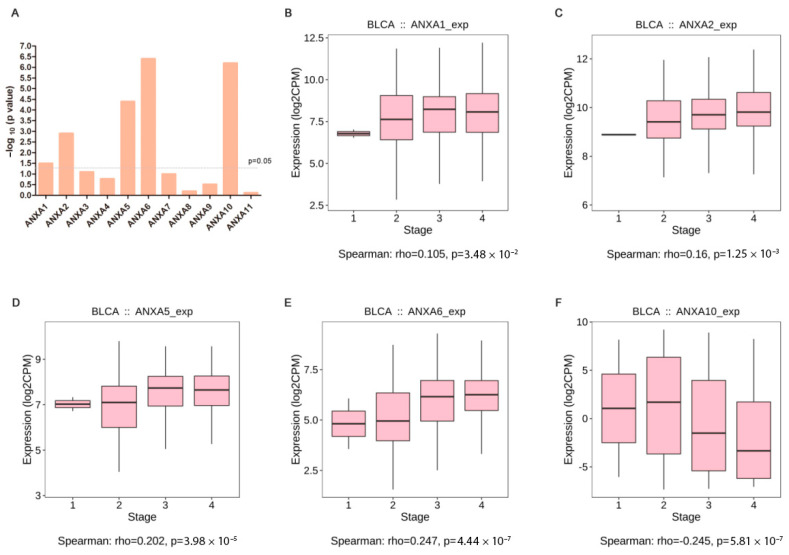
Correlation analysis of Annexin family members and clinical stages of bladder cancer in TISIDB. Summary of spearman’s correlation between Annexin family members and clinical stages (**A**); Different expression of ANXA1 (**B**), ANXA2 (**C**), ANXA5 (**D**), ANXA6 (**E**), ANXA10 (**F**) between clinical stages.

Correlation between Annexin expression and clinical stage of BC are shown in Figure 4A. The results showed that the expression levels of *ANXA1/2/5/6* were positively correlated with clinical stage, and *ANXA10* showed negative correlation with clinical stage, while no significant correlation was found in other Annexins. Additionally, high expression of *ANXA1/2/5/6* were found in BC patients with stage III/IV compared to those in BC patients with stage I/II (Figure 4B–E), whereas low expression of *ANXA10* were found in stage III/IV BC patients (Figure 4F). 

### 3.2. Construction and Validation of the Annexin-Related Prognostic Signature 

**Figure 5:**
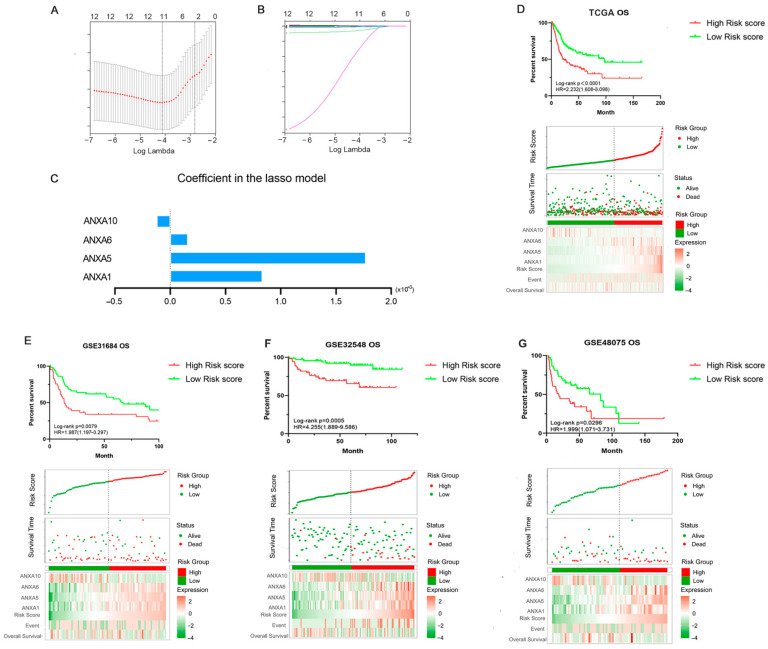
Construction and validation of Annexin-Related Prognostic Signature (ARPS). LASSO algorithm was used to construct a prognosis model (**A**–**C**); Kaplan-Meier curve, distribution diagram of risk score and survival status in TCGA BC patients (discovery cohort) between high/low-risk groups (**D**); Kaplan-Meier curve, distribution diagram of risk score and survival status in three BC validation cohorts GSE31684 (**E**), GSE32548 (**F**) and GSE48075 (**G**).

Through LASSO Cox regression, four Annexin members including ANXA1/5/6/10 were identified and used to construct Annexin-related prognostic signature (ARPS) (Figure 5A–C). Risk score of ARPS was calculated according to the formula, Risk score = 0.00083 × Exp_ANXA1_ + 0.0017 × Exp_ANXA5_ + 0.00016×Exp_ANXA6_ − 0.00012 × Exp_ANXA10_. Then, BC samples were divided into high/low-risk groups by ARPS according to the best cut-off of risk score. 

The prognostic performance of ARPS in the BC cohort was evaluated in the TCGA dataset (Discovery cohort) and validated in three independent GEO datasets (Validation cohorts, GSE31684, GSE32548, GSE48075). As shown in Figure 5D, Kaplan-Meier plot showed that BC patients in the high ARPS risk group had shorter OS time than those in the low ARPS risk group (*p* < 0.0001, HR = 2.232). The gene expression heat map indicated that high expression of *ANXA1*, *ANXA5,* and *ANXA6* but low expression of *ANXA10* were shown in the high-risk group in comparison to the low-risk group. In addition, high ARPS risk score were consistently related to short OS in GSE31684 (*p* = 0.0079, HR = 1.987, Figure 5E), GSE32548 (*p* = 0.0005, HR = 4.255, Figure 5F) and GSE48075 (*p* = 0.0296, HR = 1.999, Figure 5G). In addition, the high ARPS risk group had a shorter DSS and PFI than those with low ARPS risk in the TCGA BC cohort and GSE31684 BC cohort (Supplementary Figure S2).

Univariate and multivariate COX regression were performed to explore whether the ARPS was an independent prognostic predictor for BC. In the univariate analysis of the TCGA dataset (Discovery cohort), risk score, grade and age were all correlated with OS, and then included in subsequently multivariate analysis. Multivariate analysis showed that high ARPS risk score was associated with poor prognosis in both discovery BC cohort [(*p* < 0.0001, HR= 2.045 (1.485–2.817), Table 2] and three independent validated BC cohorts GSE31684 [*p* = 0.0010, HR= 2.259 (1.375–3.711), Table 3], GSE3254 [(*p* = 0.0060, HR = 3.591(1.453–8.872)] and GSE48075 [(*p* = 0.0100, HR = 2.291 (1.224–4.286)]. Overall, these results all confirmed that the ARPS risk score is an independent survival predictor for BC patients.

### 3.3. Associations of ARPS with Clinicopathological Features of BC

**Figure 6:**
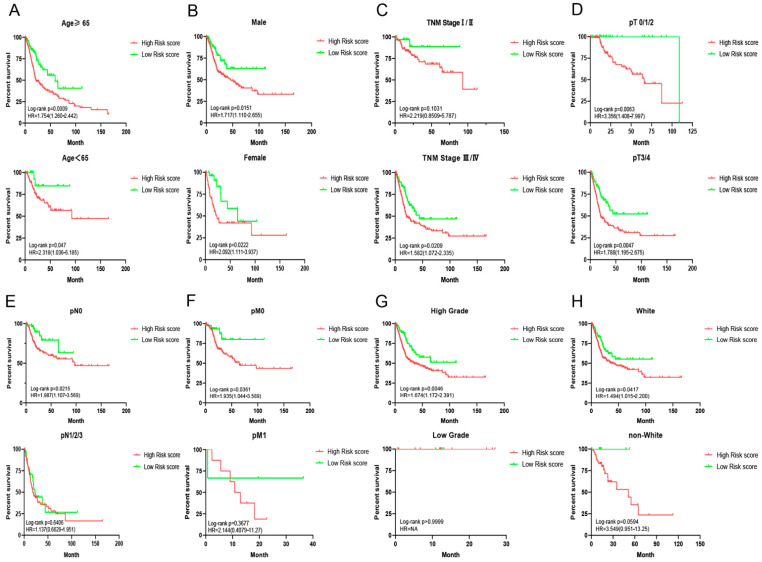
Survival analyses of ARPS in subgroup of BC patients stratified by different clinical characteristics. (**A**) Age; (**B**) gender; (**C**) TNM stage; (**D**) T stage; (**E**) M stage; (**F**) N stage, (**G**) grade and (**H**) race.

In order to better understand the role of ARPS in clinical outcomes of BC, we further investigated the relationships between ARPS and the pathological features of BC, including age, gender, grade, lymph invasion status, pT stage, pN stage, pM stage, TNM stage and race. Chi-squared test (Table 4) demonstrated that the clinicopathological features including gender, grade, pT stage, pN stage, TNM stage and race showed significant association with ARPS risk score. Further subgroup analyses were performed to determine whether ARPS could predict prognosis of BC patients under certain clinicopathological circumstances. Kaplan-Meier survival analysis (Figure 6) revealed that worse OS was noted in the high-risk ARPS groups regardless of age (Figure 6A), gender (Figure 6B), and pT stage (Figure 6D). However, ARPS is more potent to predict the outcome for higher TNM stages (Figure 6C), pN0 stage (Figure 6E), pM0 stage (Figure 6F), high grade (Figure 6G), and white (Figure 6H) than lower TNM stages, pN 1/2/3, pM 1, low grade and non-white, respectively.

### 3.4. Gene-Gene Interaction Network and Function Analysis of ARPS in BC

**Figure 7:**
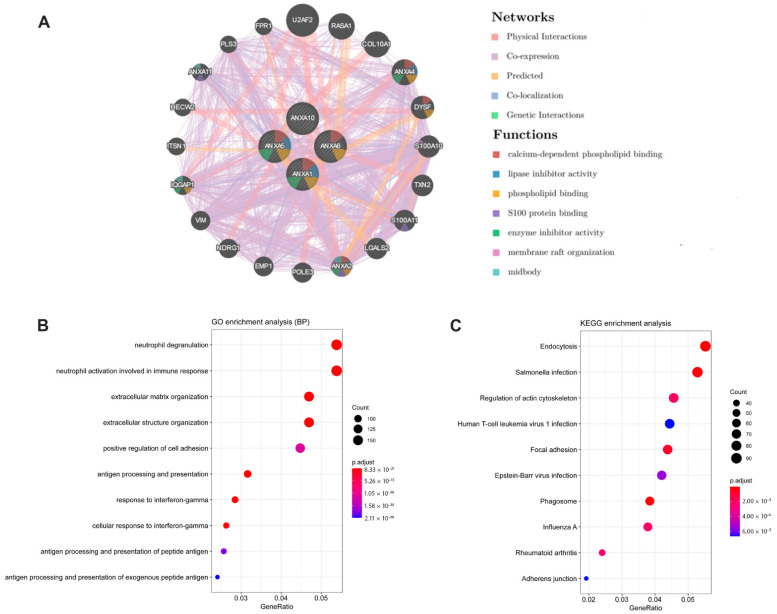
Results of gene-gene interaction network (GeneMANIA) and functional enrichment analyses between the high/low risk groups. (**A**) Gene-gene interaction network analysis of ARPS members (GeneMANIA); (**B**) GO enrichment analysis (Biological Process); (**C**) KEGG pathway analysis.

**Figure 8:**
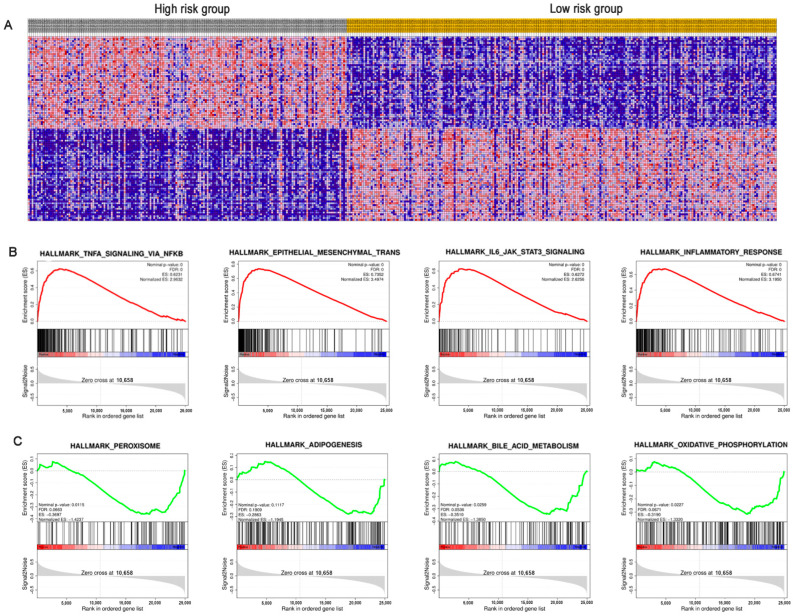
Gene-set enrichment analysis (GSEA) between the high/low-risk groups. (**A**) Heat map of differential expression genes; (**B**) significantly enriched pathways in the high-risk group; (**C**) significantly enriched pathways in the low-risk group.

A gene-gene interaction network of ARPS was constructed using the GeneMANIA database. As shown in Figure 7A, the top five genes displaying the greatest correlations with ARPS included *U2AF2*, *RASA1*, *ANXA4*, *COL10A1* and *DYSF*. Functional analysis revealed that these genes showed the greatest correlation with calcium-dependent phospholipid binding, lipase inhibitor activity, phospholipid binding S100 protein binding and enzyme-inhibitor activity. The predictive power of ARPS in predicting recurrence risk of BC patients could be attributed to their crucial roles in tumor development or metastases. Therefore, we further explored the underlying biological functions of ARPS through GO, KEGG, and GSEA pathway enrichment analyses. Gene differential analysis identified that there were 2439 differentially expressed genes (DEGs) between these two groups with high/low ARPS risk, including 1604 upregulated genes and 1710 downregulated genes. GO analysis (Figure 7B) showed that DEGs were mainly involved in cell-cell signaling (GO:0007267), immune response (GO:0006955) and chemokine-mediated signaling pathway (GO:0070098), and KEGG pathway enrichment (Figure 7C) revealed that the DEGs were mainly enriched in cytokine-cytokine receptor interaction (hsa04060), chemokine signaling pathway (hsa04062), and drug metabolism (hsa00982). Moreover, GSEA enrichment results (Figure 8A–C, Supplementary Figure S4) showed that immune related pathways such as epithelial–mesenchymal transition, IL6/JAK/STAT3 signaling, inflammatory response and TNFA signaling via NFKB were enriched in the high-risk groups (Figure 8B), while the low-risk group mainly regulated metabolism-related processes, such as adipogenesis, bile acid metabolism, oxidative phosphorylation and peroxisome (Figure 8C). 

### 3.5. Relation between ARPS and the Degree of Immune Cell Infiltration

**Figure 9:**
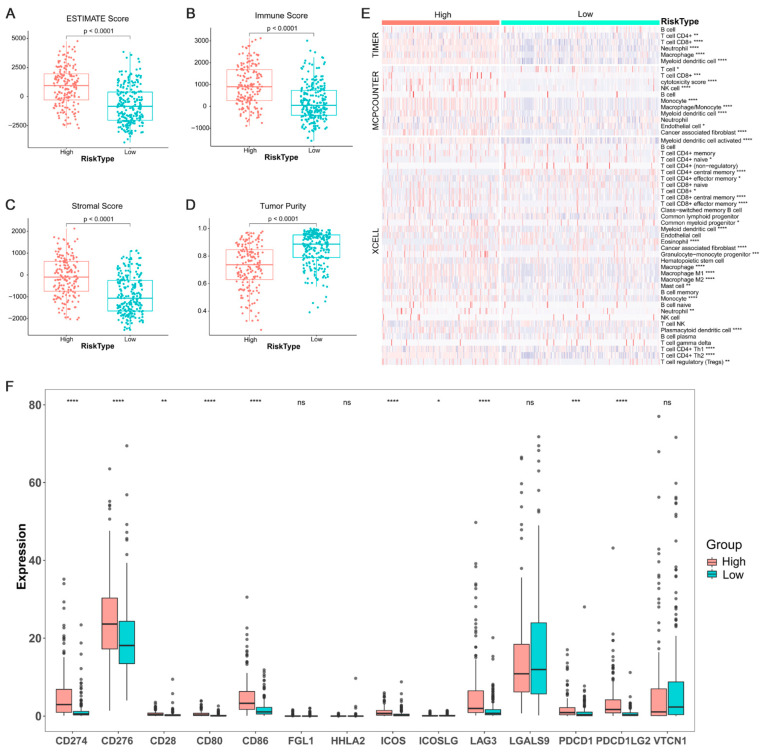
Tumor immune infiltrating and the expression of immune check gene between the high/low-risk groups. Tumor immune infiltrating level between the high/low-risk groups (**A**–**D**); proportions of tumor-infiltrating cells between the high/low-risk groups (**E**); different expression levels of immune check genes between the high/low-risk groups (**F**). Where *, *p* < 0.05, **, *p* < 0.01, ***, *p* < 0.001 and ****, *p* < 0.0001, ns not significant.

Immune cell infiltration of BC cases with high/low ARPS risk were estimated and compared by estimate algorithm (Figure 9A–D). The result showed that the risk score of ARPS was significantly positively correlated with immune infiltration level, and BC cases with high ARPS risk score had greater ESTIMATE score (Figure 9A), immune score (Figure 9B) and stromal score (Figure 9C), but unsurprisingly lower purity than those in the low ARPS risk group (Figure 9D). Through Timer, MCPcounter and xCell algorithm, we compared the immune cell abundance between the high- and low-risk groups and found that several types of immune cells, including CD8^+^ T cells, neutrophils, macrophages, myeloid dendritic cell, Tregs, and cancer-associated fibroblasts were significantly more abundant in the high-risk group than those in the low-risk group (Figure 9E). Moreover, we compared the different expression of several immune check genes between the high- and low-risk groups. The results revealed that elevated expression of most immune check genes, including *CD274*, *CD276*, *CD28*, *CD80*, *CD86*, *ICOS*, *ICOSLG*, *LAG3*, *PDCD1* and *PDCDLG2,* were found in the high-risk group compared to those in the low-risk group (Figure 9F).

## 4. Discussion

Although the advance of surgical methods and medical therapies have improved the treatment of bladder cancer, high rate of recurrence after operation and frequent metastasis lead to poor prognosis of BC patients. Identification of new prognostic biomarkers and therapeutic targets could be essential ways to improve the outcome of BC patients. In this study, we comprehensively analyzed the gene expression and prognosis of Annexin family members in BC, and constructed and validated an ARPS, which could be an independent prognostic biomarker in four individual BC cohorts. 

During our evaluation of the gene expression and prognostic value of Annexins in BC, we found that several Annexins were aberrantly expressed and associated to prognosis in BC. For example, high expression of *ANXA2/3/13* were found in BC compared to normal tissue (Table 1) and related to poor prognosis in BC patients (Figure 2, Figure 3 and Figure 4). ANXA2 is mainly distributed in the nucleus and cytoplasm, and important role in cancer progression and invasion has been reported [22]. Previous studies reported that ANXA2 was significantly elevated in tumor issues and related to poor prognosis in breast cancer [23], glioma [24], gastric cancer [25] and liver cancer [26]. ANXA3 was also reported as an important role in a variety of tumor development processes [27]. Overexpressed ANXA3 could promote tumor proliferation and metastasis in breast, lung, liver, and ovarian cancer, and was associated with chemotherapy resistance [28,29]. In addition, increased expression of ANXA13 could promote the proliferation and migration of lung cancer cells in vitro and was associated with poor survival in lung adenocarcinoma patients [30]. Moreover, Wu et al. (2021) recently reported that the expression of Annexins were related to the molecular subtypes of MIBC [31]. They found that *ANXA1*/*2*/*3*/*5*/*6*/*7*/*8* were highly expressed in basal-subtype MIBC, while *ANXA4*/*9*/*10*/*11* were mainly expressed in luminal-subtype MIBC, which might be used as potential markers for subtype classification of BC. Their results could show that the abnormal expression of Annexin members were common in several types of cancer and might play key roles in carcinogenesis and cancer progression, including BC.

We then constructed an ARPS using the machine learning algorithm LASSO and demonstrated that BC patients in the high ARPS risk group had a shorter OS/DSS/PFI in BC cohorts than those with low risk through KM-survival analysis (Figure 5). Additionally, Cox regression analysis showed that ARPS was an independent prognostic predictor in both the discovery BC cohort and three independent validation cohorts, respectively. Moreover, ARPS can even predict the prognosis of BC patients within different subgroups stratified by clinical characteristics, including age, gender and T stage. Overall, these results all confirmed that the risk score derived from ARPS could accurately and stably predict the survival outcome of BC patients independently.

KEGG pathway and GSEA analysis revealed that EMT and its regulators pathways (TGF-β signaling pathway, TNF-alpha/NF-kappaB, PI3K/AKT/mTOR) were found to be differentially enriched between the high- and low-risk groups. EMT is a process by which epithelial cells lose their epithelial properties and obtain a mesenchymal phenotype, and could transform tumor cells from inactive cancer to malignant phenotypes [27,28]. Previous studies have indicated that EMT was a key controller in tumor progression and metastasis of BC [32,33,34]. Upregulation of EMT transcription factors, such as TWIST1, ZEB1/2 and SNAI1/2 have been reported to promote migration and invasion of tumor cells in many types of tumors [35,36,37,38]. In addition, several EMT regulatory pathways, such as TNF-alpha/NF-kappaB and TGF-β were significantly highly enriched in the high-risk ARPS group. Li et al. revealed that activation of TNF-alpha/NF-kappaB could induce EMT through upregulation of EMT transcription factor Twist1 and contribute to metastatic BC [39]. Upregulation of TGF-β can activate Wnt signaling pathways and play a synergistic role to start the EMT process [40]. Moreover, the PI3K/AKT/mTOR pathway participated in numerous cell biological processes. Activated AKT and mTOR can increase E-cadherin expression and promote EMT activation [41]. Therefore, the cross-talk of these signaling pathways may contribute to the poor prognosis of the high ARPS risk group through promoting tumor recurrence and metastasis by the EMT process.

In order to escape the anti-tumor immune response, tumor cells could secrete immunosuppressive and anti-apoptotic factors or recruit suppressive immune cells to generate a highly immunosuppressive microenvironment through different mechanisms [42,43]. In BC TME, accumulated immunosuppressive cells (e.g., myeloid-derived suppressor cells (MDSCs), tumor-associated macrophages (TAMs) and regulatory T cells (T regs) and evaluated expression of immune checkpoints (e.g., CTLA-4 and PD-1) were reported to induce immune evasion of tumor cells [44,45]. Therefore, we evaluated the landscape of immune cell infiltration for the high and low ARPS risk groups by ESTIMATE, Timer, MCPcounter and xCell algorithm, which revealed that a higher degree of immune cell infiltration and greater abundance of immunosuppressive cells including Tregs, TAMs and MDSCs were found in the high ARPS risk group than these in the low ARPS risk group. Previous studies have proved that increased infiltration of Tregs, TAMs and MDSCs were found in BC tissue and were associated with poor prognosis of BC patients [46,47,48]. As key cellular components of TME, Tregs could facilitate immune evasion of cancer cells through secreting inhibitory cytokines [49], and TAMs could greatly contribute to form a tolerogenic TME by directly exhausting CD8 T cells, and supporting to traffic Tregs [50]. Additionally, MDSCs can also inhibit the immune response by suppressing CD4 T-cells, CD8 T-cells, and NK cells, inducing Tregs and facilitating TAMs polarizing into M2 phenotype [46]. Notably, MDSC-induced immunosuppression has been demonstrated to accelerate the tumor progression and enhance the formation of metastatic lesions through promoting the EMT process [51,52]. Moreover, our study suggested that the high-risk ARPS prognostic group showed high expression of *CD274*, *CD276*, *CD28*, *CD86*, *LAG3*, *PDCD1* and *PDCDLG2*, and may be more sensitive to anti-PD1 treatment. Based on above findings, we deliberate that the high-risk group might be related to a high degree of immunosuppression and low immunoreactivity in TME, thereby promoting tumor recurrence and metastasis through EMT-related pathways. As a result, the high-risk group might get more benefits from immunotherapy.

## 5. Conclusions

In conclusion, we found that several Annexins were aberrantly expressed and associated with prognosis in BC through public tools and identified and validated an ARPS comprised of four members, ANXA1/5/6/10, proving that ARPS was an independent prognostic factor in four individual BC cohorts. This model might be helpful for clinicians to guide the treatment strategy and eventually benefit BC patients. These results could also provide insights into the underlying molecular mechanisms of development and progression of BC and offer potential therapeutic targets for BC.

## Figures and Tables

**Table 1:** Comparison of mRNA expression of Annexin family members between bladder cancer and adjacent normal tissues (Oncomine database).

Gene	Datasets	Tumor (Cases Number)	Normal (Cases Number)	Fold Change	*p*-Value
ANXA1	Lee et al.	Superficial Bladder Cancer (126)	Bladder Mucosa (68)	−2.916	9.51 × 10^−14^
		Infiltrating Bladder Urothelial Carcinoma (62)	Bladder Mucosa (68)	−1.466	1.90 × 10^−2^
	Sanchez et al.	Superficial Bladder Cancer (28)	Bladder (48)	−2.131	7.07 × 10^−4^
ANXA2	Sanchez et al.	Infiltrating Bladder Urothelial Carcinoma (81)	Bladder (48)	1.493	6.23 × 10^−5^
		Superficial Bladder Cancer (28)	Bladder (48)	1.371	2.00 × 10^−3^
	Dyrskjot et al.	Infiltrating Bladder Urothelial Carcinoma (13)	Bladder (9) Bladder Mucosa (5)	2.085	4.41 × 10^−4^
ANXA3	Sanche et al.	Infiltrating Bladder Urothelial Carcinoma (81)	Bladder (48)	2.323	5.55 × 10^−8^
	Dyrskjot et al.	Infiltrating Bladder Urothelial Carcinoma (13)	Bladder (9) Bladder Mucosa (5)	2.607	2.00 × 10^−3^
ANXA4	Sanchez et al.	Infiltrating Bladder Urothelial Carcinoma (81)	Bladder (48)	1.731	3.28 × 10^−8^
		Superficial Bladder Cancer (28)	Bladder (48)	2.506	2.28 × 10^−13^
	Dyrskjot et al.	Superficial Bladder Cancer (28)	Bladder (9) Bladder Mucosa (5)	2.770	2.84 × 10^−5^
		Infiltrating Bladder Urothelial Carcinoma (13)	Bladder (9) Bladder Mucosa (5)	1.915	2.00 × 10^−3^
ANXA5	Lee et al.	Superficial Bladder Cancer (126)	Bladder Mucosa (68)	−2.392	1.01 × 10^−13^
		Infiltrating Bladder Urothelial Carcinoma (62)	Bladder Mucosa (68)	−1.417	4.00 × 10^−3^
	Sanchez et al.	Superficial Bladder Cancer (28)	Bladder (48)	−2.428	4.70 × 10^−10^
		Infiltrating Bladder Urothelial Carcinoma (81)	Bladder (48)	−1.473	6.43 × 10^−7^
	Blaveri et al.	Superficial Bladder Cancer (26)	Bladder (3)	−4.211	3.00 × 10^−3^
ANXA6	Sanchez et al.	Superficial Bladder Cancer (28)	Bladder (48)	−8.011	5.24 × 10^−25^
		Infiltrating Bladder Urothelial Carcinoma (81)	Bladder (48)	−2.846	3.69 × 10^−14^
	Dyrskjot et al.	Stage 0is Bladder Urothelial Carcinoma (5)	Bladder (9) Bladder Mucosa (5)	−1.295	4.60 × 10^−2^
		Superficial Bladder Cancer (28)	Bladder (9) Bladder Mucosa (5)	−1.558	1.00 × 10^−3^
ANXA13	Lee et al.	Infiltrating Bladder Urothelial Carcinoma (62)	Bladder Mucosa (68)	1.033	2.70 × 10^−2^
	Blaveri et al.	Infiltrating Bladder Urothelial Carcinoma (41)	Bladder (2)	2.374	8.64 × 10^−4^
		Superficial Bladder Cancer (21)	Bladder (2)	2.239	1.00 × 10^−3^

**Table 2:** Univariate and multivariate Cox analyses of ARPS risk score with OS in TCGA.

Covariates	Univariate Cox Analysis			Multivariate Cox Analysis		
	*p* Value	HR	95% CI	*p* Value	HR	95% CI
Age (>65 vs. ≤65 years)	<0.0001 ****	2.039	1.426–2.916	<0.0001 ****	1.981	1.384–2.837
Gender (Male vs. Female)	0.3460	0.846	0.598–1.198	-	-	-
Stage (III/IV vs. I/II)	<0.0001 ****	2.531	1.670–3.836	0.0010 **	2.084	1.366–3.180
Grade (High vs. Low)	0.1310	21.473	0.400–1151.678	-	-	-
Lymph (Yes vs. No)	<0.0001 ****	1.931	1.366–2.792	0.0010 **	1.843	1.271–2.671
Race (White vs. Non-white)	0.3230	1.291	0.778–2.140	-	-	-
Risk score (High vs. Low)	<0.0001 ****	2.144	1.559–2.950	<0.0001 ****	2.045	1.485–2.817

Note: Where **, *p* < 0.01 and ****, *p* < 0.0001.

**Table 3:** Univariate and multivariate Cox analyses of ARPS risk score with OS in three BC validation datasets.

Covariates	Univariate Cox Analysis						Multivariate Cox Analysis					
	GSE31684		GSE32548		GSE48075		GSE31684		GSE32548		GSE48075	
	*p* Value	HR (95% CI)	*p* Value	HR (95% CI)	*p* Value	HR (95% CI)	*p* Value	HR (95% CI)	*p* Value	HR (95% CI)	*p* Value	HR (95% CI)
Age (>65 vs. ≤65 years)	0.8710	1.047 (0.605–1.811)	0.3750	1.487 (0.619–3.569)	0.0230 *	2.371 (1.129–4.979)	-	-	-	-	0.0080 **	2.786 (1.304–5.956)
Gender (Male vs. Female)	0.9660	0.988 (0.560–1.741)	0.6300	1.273 (0.477–3.393)	-	-	-	-	-	-	-	-
Grade (High vs. Low)	0.1230	3.035 (0.741–12.43)	0.0290 *	2.790 (1.112–6.999)	-	-	-	-	0.1460	2.014 (0.783–5.182)	-	-
Stage (III/IV vs. I/II)	0.0110	2.162 (1.192–3.922)	-	-	-	-	0.0080 **	2.237 (1.230–4.068)	-	-	-	-
Risk score (High vs. Low)	0.0020 **	2.200 (1.340–3.613)	0.0010 **	4.248 (1.761–10.250)	0.0330 *	1.939 (1.056–3.559)	0.0010 **	2.259 (1.375–3.711)	0.0060 **	3.591 (1.453–8.872)	0.0100 *	2.291 (1.224–4.286)

Note: Where *, *p* < 0.05 and **, *p* < 0.01.

**Table 4:** Association of ARPS risk score with clinicopathological features in TCGA BC cohort.

Characteristics	Sample (*n* = 406)	Risk Score		χ2	*p* Value
		High Risk Score (*n* = 173)	Low Risk Score (*n* = 233)		
**Age**				2.173	0.1410
>65 years	246	112	134		
≤65 years	160	61	99		
**Gender**				4.592	0.0320 *
Male	299	118	181		
Female	107	55	52		
**Grade**				12.360	0.0004 ***
High	383	172	211		
Low	20	1	19		
**Lymph invasion**				0.582	0.4450
Yes	149	62	87		
No	130	60	70		
**TNM Stage**				6.403	0.0110 *
I-II	273	128	145		
III-IV	131	44	87		
**pT Stage**				9.258	0.0020 **
T0-T2	122	49	73		
T3-T4	251	143	108		
**pN Stage**				4.166	0.0410 *
N0	236	110	126		
N1-N3	128	74	54		
**pM Stage**				0.155	0.6940
M0	195	77	118		
M1	11	5	6		
**Race**				6.265	0.0120 *
White	323	147	176		
Non-White	66	19	47		

Note: Where *, *p* < 0.05, **, *p* < 0.01, and ***, *p* < 0.001.

## Data Availability

All of the data in this manuscript are available at TCGA (https://portal.gdc.cancer.gov/, accessed on 20 April 2018) AND GEO (https://www.ncbi.nlm.nih.gov/gds/, accessed on 20 April 2018) databases.

## Supplementary Materials

The following are available online at https://www.mdpi.com/article/10.3390/biology11020259/s1. Figure S1: The work-flow of our study, Figure S2: Comparison of mRNA expression of Annexins family members between bladder cancer and adjacent normal tissues (GEPIA2). ANXA1-11 (A-K) and ANXA13 (L); where * *p* < 0.05, Figure S3: Survival analyses of ARPS regarding Disease Free Interval (DSS) and Progression Free Interval (PFI) in TCGA (A-B) and GSE31684 (C-D) BC cohorts, Figure S4: Primary figure of Heat map of differential expression genes between the high/low risk groups (Figure 8A), Table S1: Clinical characteristics of the BC patients collected in this study.
