# Supplementary material for: Identification and Validation of an Annexin-Related Prognostic Signature and Therapeutic Targets for Bladder Cancer: Integrative Analysis
Source: Biology (Basel). 2022 Feb 7;11(2):259. doi: 10.3390/biology11020259 (PMC8869209; doi:10.3390/biology11020259)
Supplement: Supplementary file 1 [file biology-11-00259-s001.zip › biology-1529045-supplementary.pdf]

Table S1. Clinical characteristics of the BC patients collected in this study

Data source	Platform	Sample size	Age	No. of death	Media (OS)	Gender (% Male)	Stage (%I/II/III/IV/NA[a])	Survival terms
TCGA	Illumina HiSeqV2	407	69±11	155	16.93	73.71	0.49/31.70/ 34.40/32.92/0.49	OS, DSS, DFI, PFI
GSE31684	GPL570	93	69±10	65	31.31	71.12	16.13/18.28/ 45.16/20.43/0.00	OS, DSS, DFI, PFI
GSE32548	GPL6947	130	70±11	25	53.77	76.15	70.00/29.23/ 0.00/0.00/0.77	OS, DSS, DFI, PFI
GSE48075	GPL6947	73	69±10	45	30.40	-	-	OS
Total		703		290				

Note: NA[a]: Not Available; '-': no data; overall survival (OS), disease specific survival (DSS), disease free interval (DFI), and progression free interval (PFI)were defined by [1].

Reference

1. Liu, J.; Lichtenberg, T.; Hoadley, K.A.; Poisson, L.M.; Lazar, A.J.; Cherniack, A.D.; Kovatich, A.J.; Benz, C.C.; Levine, D.A.; Lee, A.V.; et al. An Integrated TCGA Pan-Cancer Clinical Data Resource to Drive High-Quality Survival Outcome Analytics. *Cell* **2018**, *173*, 400–416.e11.

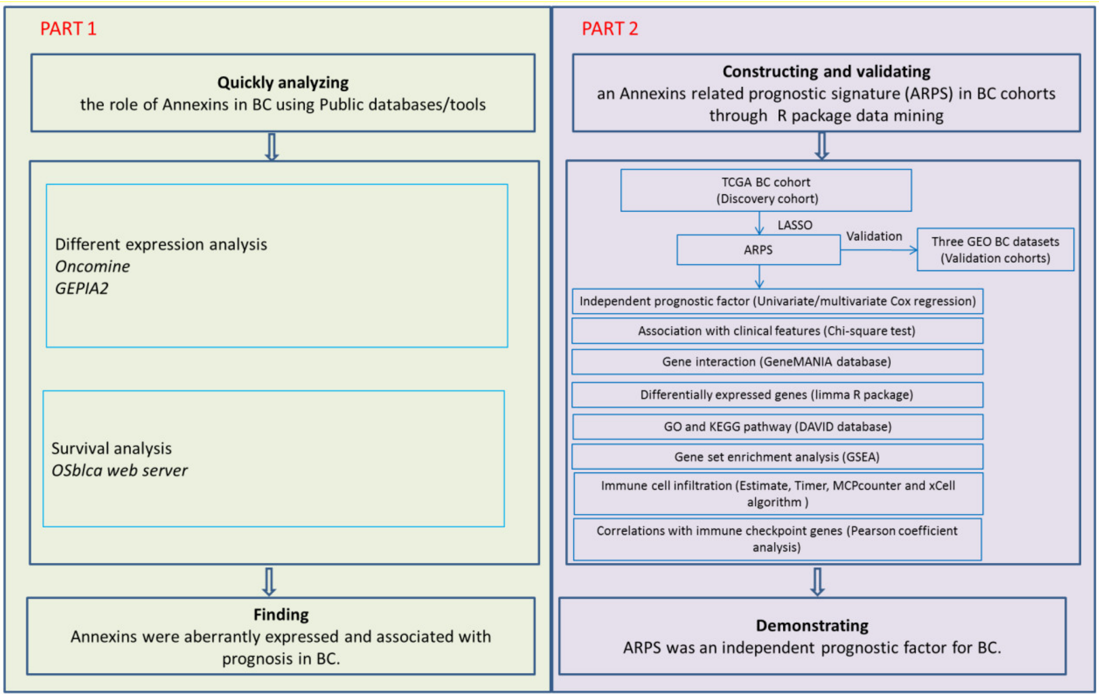

Figure S1. The work-flow of our study.

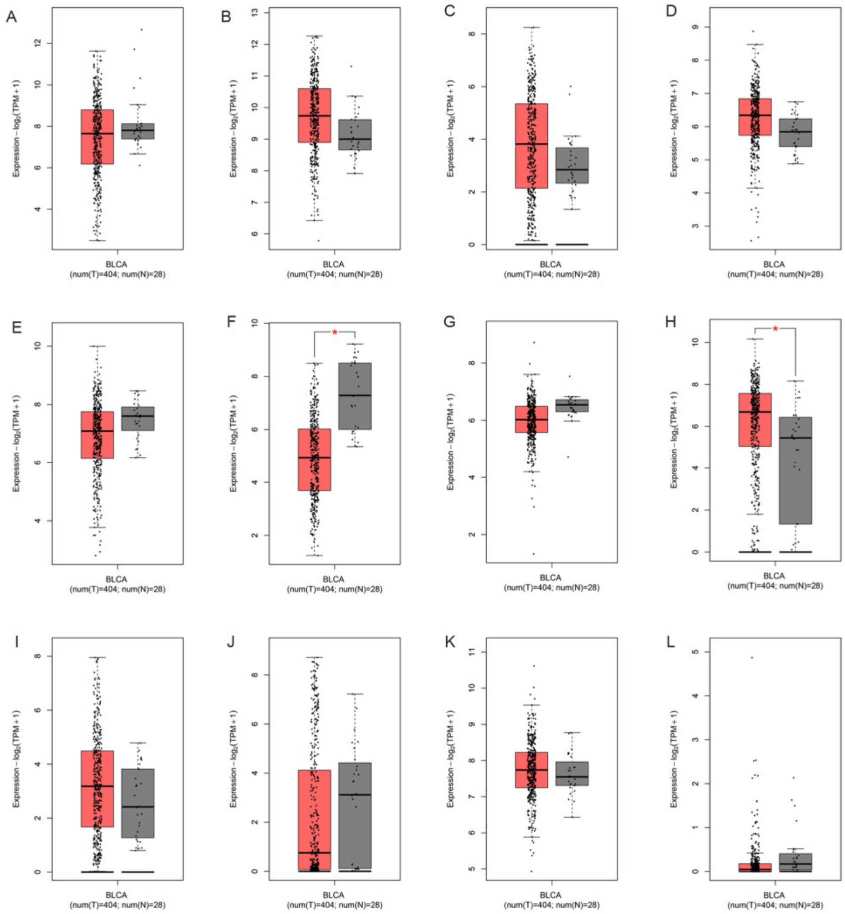

Figure S2. Comparison of mRNA expression of Annexins family members between bladder cancer and adjacent normal tissues (GEPIA2). ANXA1-11 (A-K) and ANXA13 (L); where *$p < 0.05$.

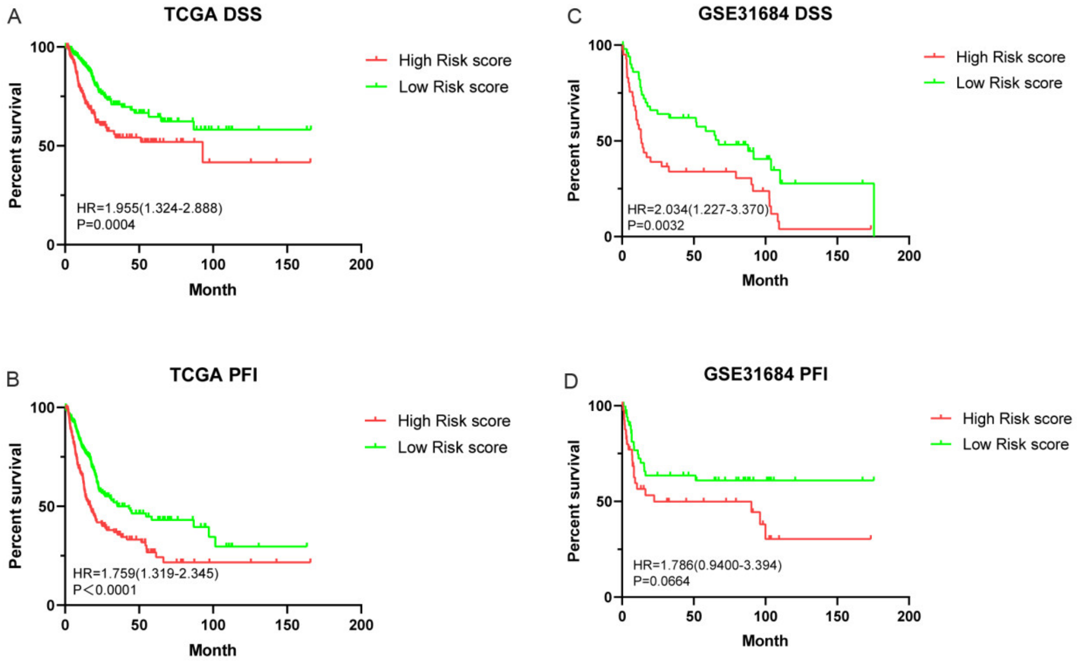

Figure S3. Survival analyses of ARPS regarding Disease Free Interval (DSS) and Progression Free Interval (PFI) in TCGA (A-B) and GSE31684 (C-D) BC cohorts.

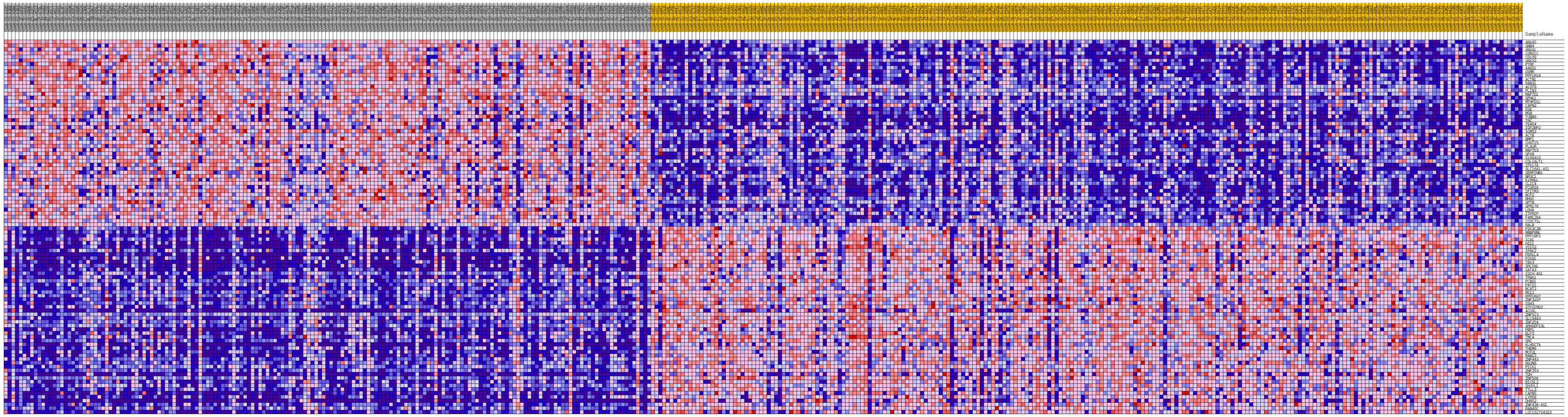

Figure S4. Primary figure of Heat map of differential expression genes between the high/low risk groups (Figure 8A).
